# Phenotypes and genotypes of mitochondrial aminoacyl‐tRNA synthetase deficiencies from a single neurometabolic clinic

**DOI:** 10.1002/jmd2.12079

**Published:** 2019-12-18

**Authors:** Aaisha Al Balushi, Diana Matviychuk, Rebekah Jobling, Gajja S. Salomons, Susan Blaser, Saadet Mercimek‐Andrews

**Affiliations:** ^1^ Division of Clinical and Metabolic Genetics, Department of Pediatrics University of Toronto, The Hospital for Sick Children Toronto Ontario Canada; ^2^ Genome Diagnostics Laboratory, Department of Pediatric Laboratory Medicine University of Toronto, The Hospital for Sick Children Toronto Ontario Canada; ^3^ Metabolic Laboratory, Department of Clinical Chemistry VU University Medical Center Amsterdam The Netherlands; ^4^ Neuroscience Campus Amsterdam The Netherlands; ^5^ Division of Neuroradiology, Department of Diagnostic Imaging University of Toronto, The Hospital for Sick Children Toronto Ontario Canada

**Keywords:** *DARS2*, *EARS2*, global developmental delay, leukodystrophy, mitochondrial aminoacyl‐tRNA synthetase deficiencies, movement disorders, *PARS2*, *RARS2*, seizures

## Abstract

Mitochondrial aminoacyl‐tRNA synthetases play a major role in protein translation, synthesis, and oxidative phosphorylation. We reviewed all patients diagnosed with mitochondrial aminoacyl‐tRNA synthetase deficiencies diagnosed in a single neurometabolic clinic. We report five patients with mitochondrial aminoacyl‐tRNA synthetase deficiencies including *DARS2*, *EARS2*, *PARS2*, and *RARS2* deficiencies. Siblings with *DARS2* deficiency presented with global developmental delay within the first year of life. *DARS2*, *EARS2*, *PARS2*, and *RARS2* deficiencies were identified by whole exome sequencing. We report coagulation factor abnormalities in *PARS2* deficiency for the first time. We also report symmetric increased signal intensity in globus pallidi in FLAIR images in brain MRI in *EARS2* deficiency for the first time. One patient with *RARS2* deficiency had compound heterozygous variants in *RARS2*. One of those variants was an intronic variant. We confirmed the pathogenicity by mRNA studies. Mitochondrial aminoacyl‐tRNA synthetase deficiencies are diagnosed by molecular genetic investigations. Clinically available non‐invasive biochemical investigations are non‐specific for the diagnosis of mitochondrial aminoacyl‐tRNA synthetase deficiencies. A combination of brain MRI features and molecular genetic investigations should be undertaken to confirm the diagnosis of mitochondrial aminoacyl‐tRNA synthetase deficiencies.

## 1 INTRODUCTION

Mitochondrial aminoacyl‐tRNA synthetases are a group of catalytic enzymes. They play a major role in protein translation by delivering amino acids to the nascent polypeptide chain. They contribute to the accuracy of protein synthesis and protein expression. They also contribute to the functions of oxidative phosphorylation enzymes. There are 19 mitochondrial aminoacyl‐tRNA synthetase genes.^1^ Biallelic variants in 16 of these genes have been associated with human disease including (each gene and phenotype OMIM numbers are listed in the brackets) *AARS2* (OMIM*#*612035, OMIM#614096, 615 889), *CARS2* (OMIM#612800, OMIM#616672), *DARS2* (OMIM*#*610956, OMIM#611105), *EARS2* (OMIM#612799, OMIM#614924), *FARS2* (OMIM#611592, OMIM#614946, 617 046), GARS (OMIM# 600287, OMIM#600794), *HARS2* (OMIM#600783, OMIM#614926), *KARS* (OMIM#60142, OMIM#613641), *LARS2* (OMIM#604544, OMIM#617021, 615 300), *MARS2* (OMIM#609728, OMIM#616430, 611 390), *NARS2* (OMIM#612803, OMIM#616239), *RARS2* (OMIM#611524, OMIM#611523), *SARS2* (OMIM#612804, OMIM#613845*)*, *TARS2* (OMIM#612805, OMIM#615918*) VARS2* (OMIM#611390, OMIM#615917), *YARS2* (OMIM#610957, OMIM#613561). The clinical phenotypes are variable. Neurological features include severe early onset central nervous system disease, sensorineural hearing loss and leukodystrophy in brain magnetic resonance imaging (MRI). Additionally, liver, kidney, eye and cardiac organ system involvements are reported. Currently, diagnosis of mitochondrial aminoacyl‐tRNA synthetase deficiencies are based on the molecular genetic investigations. Characteristic brain MRI features may suggest *DARS2*, *PARS2* and *RARS2* deficiencies. Whole exome sequencing will likely identify more patients to increase our knowledge of mitochondrial aminoacyl‐tRNA synthetase deficiencies in the future.

Here we report five patients from four unrelated families with four different mitochondrial aminoacyl‐tRNA synthetase deficiencies including *DARS2* (two siblings), *EARS2*, *PARS2* and *RARS2* deficiencies. The interpretation of pathogenicity of variants of unknown significance can be difficult. To assist physicians in confirming their patients’ genetic diagnostic confirmation, we describe phenotypes, genotypes and neuroimaging features of new patients with mitochondrial aminoacyl‐tRNA synthetase deficiencies.

## 2 MATERIALS AND METHODS

The Institutional Research Ethics Board approved this retrospective cohort study (Approval# 1000061126). Additionally, all families signed institutional case report consent forms. We included all patients with confirmed molecular genetic diagnoses of mitochondrial aminoacyl‐tRNA synthetase deficiencies from a single neurometabolic clinic. This is a retrospective chart review study.

We reviewed electronic patient charts for clinical features, biochemical investigations, molecular genetic tests, brain MRI and brain magnetic resonance spectroscopy (MRS) results. Whole exome sequencing or targeted Sanger sequencing of *DARS2* was performed according to molecular genetic laboratories' methods. We applied the recommendations for mutation nomenclature (http://www.hgvs.org/mutnomen) to name gene variations. We classified all variants using the Alamut variant interpretation software version 2.7‐2 (http://www.interactive-biosoftware.com). We applied in silico analysis tools including Mutation Taster NCBI 37/Ensembler 69 (MutTaster),^2^ Sorting Tolerant From Intolerant (SIFT) 4G annotator version 4.0.3 (Alamut variant interpretation software, http://www.interactive-biosoftware.com),^3^ Polymorphism Phenotyping version 2.1.0 (PolyPhen‐2)^4^ and conservation species (Alamut variant interpretation software version 2.7‐2). We also searched all variants in the Genome Aggregation Database (gnomAD) (http://gnomad.broadinstitute.org/about) for their allele frequency in the general population.^5^ We applied The American College of Medical Genetics and Genomics (ACMG) variant classification guidelines.^6^

## 3 RESULTS

There were five patients with four different mitochondrial aminoacyl‐tRNA synthetase deficiencies including *DARS2* (n = 2, two siblings, older sibling reported previously in^7^), *EARS2* (n = 1), *PARS2* (n = 1) and *RARS2* (n = 1) deficiencies. Four patients had whole exome sequencing including two research and two clinical whole exome sequencing. One patient had targeted Sanger sequencing of *DARS2* (due to positive family history). About 200 patients underwent research or clinical whole exome sequencing in this neurometabolic clinic since 2014. These patients were the only patients diagnosed with mitochondrial aminoacyl‐tRNA synthetase deficiencies in this neurometabolic clinic. The senior author received whole exome sequencing results from the research teams or clinical diagnostic laboratories with a single confirmatory genetic diagnosis.

**Figure 1:**
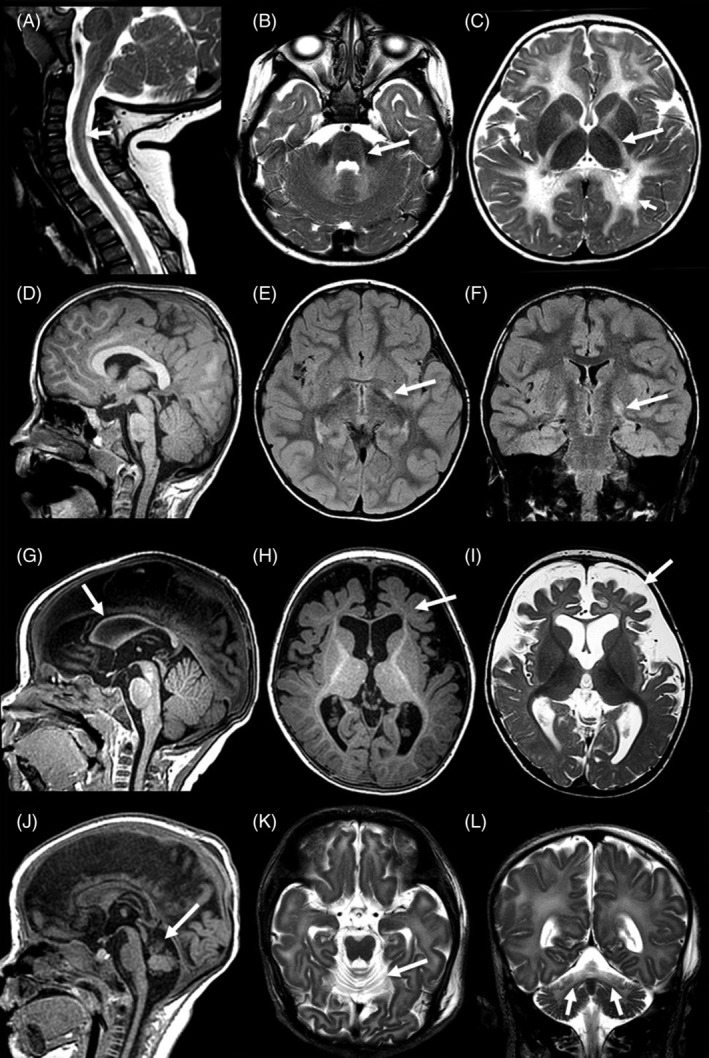
Brain MRI images of four patients with mitochondrial aminoacyl‐tRNA synthetase deficiencies were depicted. A,B,C, Brain MRI of the patient (patient 2) with *DARS2* deficiency at the age of 56 week. T2 weighted sagittal image, A, of the spine showed increased signal intensity in the dorsal column (arrows). T2 weighted axial image, B, demonstrated increased signal intensity in the cerebellar white matter (short arrow) and in the superior cerebellar peduncles white matter (arrow). Axial T2 weighted image, C, revealed involvement of the subcortical and periventricular white matter and the external capsule, anterior and posterior (arrow) limbs of the internal capsules. D,E,F, Brain MRI of the patient with *EARS2* deficiency at the age of 4 years 6 months old. There was bilateral symmetric increased signal in globus pallidus (arrows) on axial, E, and coronal, F, in FLAIR images. G,H,I, Brain MRI of the patient with *PARS2* deficiency at the age of 8 months. Sagittal T1 weighted image, G, demonstrated a markedly thinned corpus callosum. Incomplete myelin maturation of the frontal lobes was shown in axial T1 weighted image, H. Axial T2 weighted image revealed prominent frontal, I, and Sylvian CSF spaces and widened cortical sulci and anterior horns of the lateral ventricles reflecting frontal lobe volume loss. J,K,L, Brain MRI of the patient with *RARS2* deficiency at the age of 6 weeks. Superior vermian atrophy (arrow) was demonstrated in sagittal T1 weighted image. J, Marked superior vermian and superior cerebellar atrophy (arrows) was shown in axial, K, and coronal, L, T2 weighted images

Demographics, clinical features and genetic diagnosis of all patients were summarized in Table 1. Parents of patients with *DARS2* and *EARS2* deficiencies were consanguineous. All patients had normal plasma amino acids, homocysteine, acylcarnitine profile, total and free carnitine and urine organic acid analysis. In silico analysis results of all variants and their ACMG variant classification results were listed in Table 2. Brain MRS showed normal lactate peak in all patients. Brain MRI images of four patients were depicted in Figure 1. Brain MRI images of the older sibling with *DARS2* deficiency was previously reported.^7^

**Table 1:** Clinical, biochemical and molecular genetic results of all patients with mitochondrial aminoacyl‐tRNA synthetase deficiencies

	Patient1	Patient 2	Patient 3	Patient 4	Patient 5
Gender	Male	Female	Female	Female	Female
Diagnosis	*DARS2* deficiency	*DARS2* deficiency	*EARS2* deficiency	*PARS2* deficiency	*RARS2* deficiency
Current age	8 y	1.5 y	6 y	3.3 y	0.2 (passed away) y
Age of diagnosis	52 mo	11 mo	60 mo	33 mo	1.5 mo
Consanguinity	Yes	Yes	Yes	No	No
Neonatal period	Normal	Jaundice	Preterm	Normal	Encephalopathy
Age at onset	11 mo	6 mo	2 mo	4 mo	Birth
Early development	Motor delay	N	GDD	GDD	NA
Symptoms at presentation	GDD and nystagmus	Motor regression	GDD	Seizures	Hypotonia, seizures
Muscle tone	Mild axial hypotonia, peripheral hypertonia	Mild axial hypotonia, peripheral hypertonia	Axial hypotonia, peripheral hypertonia	Peripheral hypertonia	Generalized hypotonia
Seizure	No	No	No	Infantile spasm (4 mo)	Status epilepticus (1 mo)
Movement disorder	No	No	Dystonia	Bradykinesia, ataxia	No
Cerebellar signs	Nystagmus	Nystagmus	None	None	None
Microcephaly	No	No	Yes	No	No
Other	None	None	FTT	None	None
Outcome	Motor delay	Severe GDD	Severe GDD	Severe GDD	Deceased (2 mo)
Plasma lactate	Normal	Normal	3.8 mmol/L (<2.5 mmol/L)	Normal	Normal
Echocardiography	Normal	Normal	Normal	Normal	Hypoplastic pulmonary artery
Liver function	Normal	Normal	AST = 59 U/L (ref < 34 U/L)	PTT = 44 s (ref = 24‐36)	AST = 74 U/L (ref < 34 U/L)
Brain MRI/MRS	Hypomyelination of supratentorial and infratentorial and cervical spinal cord WM/normal	Increased signal in cerebral WM/normal	Increased signal in globus pallidi/normal	Cerebral atrophy, Bifrontal subdural hematoma/normal	Increased signal in WM/normal
Variants	Hmz p.Glu255Leu (c.766A>C)	Hmz p.Glu255Leu (c.766A>C)	Hmz p.Ala98Val (c.293 C>T)	Cmp Htz p.Pro364Arg (c.1091C>G)/p.Val95Ile (c.283G>A)	Cmp Htz p.Glu212Glnfs*7 (c.633_636delAGAA)/c.1113‐21A>C

Abbreviations: Cmp, compound; FTT, failure to thrive; GDD, global developmental delay; Hmz, homozygous; Htz, heterozygous; MRI, magnetic resonance imaging; MRS, magnetic resonance spectroscopy; ND, not done; WM, white matter.

**Table 2:** In silico analysis of variants in patients with inherited metabolic disorders and other genetic causes of pediatric tRNA related disorders

Gene	Variants	SIFT	MutTaster	PolyPhen‐2	Conservation in species	gmAD allele count in allele number	ACMG variant classification
*DARS2* *NM_001349.3*	c.766A>C (p.Met256Leu)	Tolerated	Disease causing	Possibly damaging	NA	NA	VUS (PM2, PP5)
*EARS2* *NM_001083614.1*	c.293C>T (p.Ala98Val)	Tolerated	Disease causing	Possibly damaging	Ten out of 13	NA	VUS (PM2, PP3, PP5)
*PARS2* *NM_152268.3*	c.1091C>G (p.Pro364Arg)^12^	Deleterious	Disease causing	Probably damaging	Eleven out of 11	Two‐hundred and ninety‐five in 281 922	Likely pathogenic (PM1, PM3, PP3, PP5)
	c.283G>A (p.Val95Ile)	Tolerated	Disease causing	Benign	Seven out of 11	Thirty‐one in 282 762	Likely pathogenic (PS4, PM2)
*RARS2* *NM_020320.4*	c.633_636delAGAA (p.Glu212Glnfs*7)	NA	NA	NA	NA	Three in 282 832	Likely pathogenic (PVS1, PM2)
	c.1113‐21A>C (r.spl?)	NA	NA	NA	NA	NA	VUS (PM2, PP3)

Abbreviations: gnomAD, Genome Aggregation Database (gnomAD); Mod, moderate; NA, not available; SIFT, sorting intolerant from tolerant; VUS, variant of unknown significance.

Siblings with *DARS2* deficiency presented with global developmental delay within the first year of life. Younger sibling had history of motor regression after a viral infection at the time of initial presentation. Additionally, older sibling had nystagmus and ataxia. In both siblings with *DARS2* deficiency, the brain MRI revealed increased signal intensity in the dorsal columns, inferior and superior cerebellar peduncles, cerebellar white matter, the external and internal capsules and subcortical and periventricular white matter in T2 weighted images.

The patient with *EARS2* deficiency had global developmental delay, generalized dystonia and dyskinesia within the first year of life. She had mild aspartate aminotransferase (AST) elevation. Plasma lactate level was mildly elevated (3.8 mmol/L; reference ranges <2.5 mmol/L) as well. Her brain MRI revealed symmetric increased signal intensity of the globus pallidus in FLAIR images.

The patient with *PARS2* deficiency had seizures, nystagmus, ataxia, generalized dystonia and acquired microcephaly. She had coagulation abnormalities including prolonged partial thromboplastin time (PTT), and low factor IX and XI levels. Her brain MRI revealed markedly thinned corpus callosum in T1 weighted images and volume loss of the cortical and subcortical frontal lobes, and widening of the cortical sulci, Sylvian fissures and frontal horns in T2‐weighted images. There was a transient delay in myelin maturation in T1‐weighted brain MRIs performed at the age of 4 and 8 months, which was normalized at the age of 25 months.

The patient with *RARS2* deficiency presented with neonatal epileptic encephalopathy in the first day of life. She was diagnosed at the age of 1.5 months. She had a hypoplastic proximal pulmonary artery branch in echocardiography. She had mild AST elevation. Her brain MRI revealed superior vermian and superior cerebellar atrophy in the T1 weighted images at the age of 5 days. Her repeat brain MRI showed a mild dilatation of the anterior horns of the lateral ventricles suggestive of a progressive supratentorial volume loss in T2 weighted images at the age of 6 weeks. She passed away at the age of 2 months.

Patients with *DARS2*, *EARS2*, and *PARS2* deficiencies had either homozygous or compound heterozygous missense variants. Missense variants in *DARS2* and *EARS2* were classified as variant of unknown significance by ACMG variant classification guidelines. Both variants were classified as disease causing and possibly damaging in two out three in silico prediction tools and both of them were not reported in public databases in the general population or as polymorphisms in the dbSNP database. Variant in *DARS2* resulted in the similar phenotypic and brain MRI features in both siblings. Variant in *EARS2* was highly conserved in species. Patient with *RARS2* deficiency had two novel truncating variants, c.633_636delAGAA (p.Glu212Glnfs*7) and c.1113‐21A>C in *RARS2*. To investigate pathogenicity of the intronic variant, RNA was isolated from lymphoblasts and reverse transcriptase PCR and Sanger sequencing of the *RARS2* cDNA were performed. A deletion r.633_636delAGAA was detected and a splice error harboring a deletion of exon 14 (r.1113_1273del). Parental testing confirmed the carrier status in the parents in all patients.

## 4 DISCUSSION

We report the phenotypes and genotypes of five patients with mitochondrial aminoacyl‐tRNA synthetase deficiencies from a single neurometabolic clinic to expand our current knowledge for these disorders. Gross motor delay with relatively normal cognitive functions and spasticity were prominent features in patients with *DARS2* deficiency, while the patients with *EARS2* and *PARS2* deficiencies had cognitive dysfunction and movement disorders. Seizures were well controlled with a single anti‐epileptic medication in patients with *EARS2* and *PARS2* deficiencies. Mild elevation of AST was present in patients with *EARS2* and *RARS2* deficiencies and synthetic liver dysfunction was present in the patient with *PARS2* deficiency. To the best of our knowledge, we report for the first time prolonged PTT, and low factor IX and XI levels in *PARS2* deficiency.

*DARS2* deficiency has been first reported as leukoencephalopathy with brainstem and spinal cord involvement in brain MRI and elevated lactate in brain MRS in 2007.^8^ More than 75% of patients had elevated lactate in brain MRS. Serum and cerebrospinal fluid lactate levels were elevated in less than 20% of patients. Only one family with three affected children were reported with an intrafamilial phenotypic variability. Two younger siblings had infantile onset global developmental delay and the oldest sibling presented with a mild lower limb spasticity at the age of 20 years. The older sibling was diagnosed due to the diagnosis of the younger siblings.^9^ In our study, both siblings with *DARS2* deficiency had global developmental delay within the first year of life. Younger sibling presented with motor regression after a viral infection at the age of 6 months.

*EARS2* deficiency has been first reported as leukoencephalopathy with thalamus and brainstem involvement in brain MRI and elevated lactate in brain MRS in 2012.^10^ Seizures were reported in 15% of patients. Infantile spasms were the most common seizure type. Elevated serum lactate levels were reported in 15% of patients ranging from 2.6 to 7.4 mmoL/L. Two patients had intermittent elevation of liver enzymes up to five times. Brain MRI features included symmetric signal changes in the deep white matter (usually sparing the periventricular rim), thalami and brainstem. Increased lactate in brain MRS was also reported.^11^ One patient had typical brain MRI features at the age of 8 months, which partially improved at the age of 29 months. A repeat brain MRI demonstrated new increased signal intensity in the left caudate and left globus pallidus in T2 weighted images at the age of 5 years and 10 months.^12^ Our patient had low apgar scores at birth and was resuscitated at the time of delivery. Based on this history, she was diagnosed with hypoxic‐ischemic encephalopathy. Her brain MRI showed bilateral increased signal intensity in globus pallidus in FLAIR images, but there were no neuroimaging features of hypoxia or other typical MRI features of *EARS2* deficiency. To the best of our knowledge, we report isolated bilateral globus pallidus changes in FLAIR images in brain MRI in *EARS2* deficiency for the first time.

*PARS2* deficiency was first described by Sofou et al.^13^ The phenotypic spectrum ranged from infantile onset neurodegenerative disease, called Alpers syndrome, to global developmental delay and ataxia. All patients had infantile onset seizures. Infantile spasms were the most common seizure type in more than 60% of patients. Hepatocellular dysfunction was reported in few patients with *PARS2* deficiency.^13^, ^14^ None of the patients had synthetic liver dysfunction in that study. To the best of our knowledge, we report synthetic liver dysfunction characterized by prolonged PTT, low factor IX and XI levels for the first time. Typical brain MRI features included leukoencephalopathy, cerebral atrophy especially in the perisylvian and fronto‐temporal brain regions and abnormal signal intensities in the basal ganglia.^15^, ^16^ Interestingly, perisylvian and fronto‐temporal atrophy in brain MRI resembles brain MRI features of glutaric aciduria type I. We think that it is important to include *PARS2* deficiency in the differential diagnosis of perisylvian and fronto‐temporal brain atrophy in brain MRI in addition to glutaric aciduria type 1.

*RARS2* deficiency was first described as pontocerebellar hypoplasia 6 disease by Edvardson et al.^17^ The majority of patients with *RARS2* deficiency had pontocerebellar hypoplasia or progressive cerebral and pontocerebellar atrophy.^18^ About one‐third of the variants in *RARS2* are truncating variants.^18^ Interestingly, we also identified compound heterozygous truncating variants, a 4 base pair deletion and an intronic variant in *RARS2*. Based on the phenotype and suggestive neuroimaging features as well as functional studies, we confirmed the diagnosis of *RARS2* deficiency. We report for the first time an intronic variant in *RARS2*.

Reports of variant of unknown significance in molecular genetic investigations pose difficulties for clinicians. Identification of these variants require extensive phenotypic and neuroimaging analysis as well as application of in silico analysis tools to confirm a genetic diagnosis. Functional analysis of variants in mitochondrial aminoacyl‐tRNA synthetase genes will be essential to guide clinicians for their diagnostic odyssey in the future.

In summary, we report five patients with mitochondrial aminoacyl‐tRNA synthetase deficiencies from a single neurometabolic clinic. Combination of characteristic brain MRI features and molecular genetic investigations should be undertaken to confirm the diagnosis of mitochondrial aminoacyl‐tRNA synthetase deficiencies.

## CONFLICT OF INTEREST

The authors declare no potential conflict of interest.

## AUTHOR CONTRIBUTIONS

A.A.B. reviewed charts, generated database, drafted the manuscript, conducted the work, approved the final version. D.M. reviewed all variants for in silico analysis and classified all variants based on the ACMG variant classification guidelines, approved the final version. R.J. provided whole exome sequencing analysis for the patient with *RARS2* deficiency and approved final version of the manuscript. G.S.S. provided functional analysis for *RARS2* variants and approved final version of the manuscript. S.B. reviewed the brain MRIs and prepared figures and approved the final version of the manuscript. S.M.‐A. provided planning, conduct, drafting and revising the manuscript, and approved the final version of the manuscript. S.M.‐A. reported the work described in the article, who serves as guarantor for the article, accepts full responsibility for the work and/or the conduct of the study, had access to the data, and controlled the decision to publish.

## INFORMED CONSENT

Institutional Research Ethics Board (Approval# 1000061126) approved this study. Additional informed consent was obtained from parents for all patients for which identifying information is included in this article. All procedures followed were in accordance with the ethical standards of the responsible committee on human experimentation (institutional and national) and with the Helsinki Declaration of 1975, as revised in 2000 (5). Informed consent was obtained from all patients for being included in the study. Proof that informed consent was obtained is available upon request.

## ANIMAL RIGHTS

This article does not contain any studies with animal subjects performed by the any of the authors.

## Data Availability

The authors confirm that the part of the data supporting the findings of this study are available within the article (and/or) its Supporting Information based on the Research Ethics Board approvals. The data that support the findings of this study are available on request from the corresponding author. The data are not publicly available since it contains research participants’ information that could compromise the privacy of the research participants.
